# A Cross-Sectional Study Into the Prevalence of Dairy Cattle Lameness and Associated Herd-Level Risk Factors in England and Wales

**DOI:** 10.3389/fvets.2018.00065

**Published:** 2018-04-05

**Authors:** Bethany E. Griffiths, Dai Grove White, Georgios Oikonomou

**Affiliations:** ^1^Department of Livestock Health and Welfare, Institute of Veterinary Science, University of Liverpool, Neston, United Kingdom; ^2^Institute of Ageing and Chronic Disease, University of Liverpool, Liverpool, United Kingdom; ^3^Department of Epidemiology and Population Health, Institute of Infection and Global Health, University of Liverpool, Neston, United Kingdom

**Keywords:** dairy cattle, lameness, risk factors, claw trimming, housing

## Abstract

Lameness is one of the most pressing issues within the dairy industry; it has severe economic implications while causing a serious impact on animal welfare. A study conducted approximately 10 years ago found the within farm lameness prevalence in the UK to be 36.8%. Our objective here is to provide an update on within farm lameness prevalence in the UK, and to provide further evidence on farm level risk factors. A convenience sample of 61 dairy farms were recruited across England and Wales from September 2015 to December 2016. A single farm visit was made and the milking herd was mobility scored, as the cows exited the milking parlor after morning, afternoon, or evening milking. Information regarding the farm and management system was then collected using a short interview with the farmer followed by collection of various subjective and objective measurements of the environment. The same, trained researcher performed all animal and facility-based measures on all visits. A series of univariable analyses were conducted to evaluate the association between various risk factors and herd lameness prevalence (logit transformed). A multivariable linear regression model was then fitted. The median number of milking cows per herd was 193, ranging from 74 to 1,519 cows. The mean within farm lameness prevalence was 31.6%, ranging from 5.8 to 65.4%. In total, 14,700 cows were mobility scored with 4,145 cows found to be lame (28.2%). A number of risk factors were associated with lameness at the univariable analysis level. Categorical risk factors retained in the final model were: resting area type, collecting yard groove spacing width, whether farms were undertaking the 60- to 100-day post calving claw trimming and the frequency of footbathing in the winter. The amount of concentrates fed in the milking parlors or out of parlor feeders was also associated with lameness prevalence. The results of this study have provided an update on the UK herd lameness prevalence and have confirmed the importance of cow comfort and footbathing frequency. The association between early lactation claw trimming and reduced lameness prevalence is, to the best of our knowledge, reported for the first time.

## Introduction

Lameness is considered to be one of the most pressing issues within the dairy industry today. It is described as a clinical symptom, recognizable as impaired locomotion, usually associated with lesions of the hind limb (1), and with more than 90% of lesions found in the foot (2). Lameness causing lesions can be of both infectious and non-infectious etiology. The main lameness causing infectious lesions are digital dermatitis and interdigital phlegmon (foul in the foot), while the main non-infectious lesions are sole ulcers and white line disease (3). Lameness severely affects welfare, fertility, and milk yield leading to considerable economic losses (4). In a study conducted approximately 10 years ago, the cost of lameness to the UK industry was calculated to be £127,822,855 per annum (5). In 2010, the mean within farm prevalence of lameness in the UK was estimated at 36.8% (6); this represented a 16.2% increase from the prevalence estimated in 1996 (2). However, lameness issues are not confined to the UK, worldwide estimates have estimated the mean within farm prevalence to range from 14 to 31% (7–10).

Lameness is multifactorial in nature and as such a wide array of on-farm risk factors have been identified in many studies both within the UK and worldwide. Larger herd size has been identified as a risk factor (11), however, other studies have not supported this finding (9, 12). The length of the housing period has also been shown to be a risk factor (6, 12–15). Deep bedded cubicles have been associated with reduced lameness prevalence in a number of studies (6, 11, 16, 17). It is thought that increased bedding depth is positively associated with cow comfort, resulting in greater cubicle occupancy and reduced standing times (18, 19), a well-defined risk factor for lameness (20–22). Straw yards are also associated with increased lying comfort and, therefore, reduced lameness prevalence (6, 15, 23). Deep bedded cubicles and well managed straw yards could also be associated with better hygiene and, therefore, decreased infection pressure and decreased incidence of lameness causing lesions of infectious etiology. Other factors resulting in increased standing times, such as overstocking (24) or poor cubicle design (6, 15, 16, 25), will also increase the risk of lameness (21).

Routine preventative claw trimming has been linked to a decrease in lameness prevalence in several studies (13, 25–27), although Barker et al. (28) was unable to replicate these results and instead found it to be a risk factor for increased lameness. The provision of a commercial claw trimmer, and failure to wash claw trimming equipment between cows was found to be associated with increased lameness prevalence in several studies (15, 29). Furthermore, an appropriate use of footbathing was found to decrease the risk of lameness (15, 23, 27) as was treating lame cows within 48 h of detection of lameness signs.

Lameness prevalence and associated risk factors for UK dairy herds were last examined in 2010 (6). Since then, DairyCo [now Agriculture and Horticulture Development Board (AHDB) Dairy], a levy funded, non-profit organization working on behalf of British dairy farmers, launched the Healthy Feet Program. The aim of this program was to help farmers decrease the lameness prevalence within their herds (30). Furthermore, key industry stakeholders have placed greater emphasis on decreasing lameness within UK dairy herds. Given the increased attention to this issue, it would be expected that lameness prevalence within UK herds should be decreasing; however, this is yet to be shown. Given the high prevalence, extensive, and severe effects and the recent efforts to decrease lameness prevalence within UK herds, the aim of this study is to provide an update on lameness prevalence, and to describe the farm level risk factors encountered on these farms.

## Materials and Methods

### Farm Recruitment and Visits

A convenience sample of 61 farms was recruited with the help of three veterinary practices and a dairy consultant working nationally. An initial telephone call was made to 124 farmers; 61 of them expressed an interest in participating in the study; 63 of the approached farmers were not interested in participating in the study. The number of approached and eventually visited farms was mainly dictated by the limited funds and time available for this study. Farms featuring robotic milking facilities were excluded, as were organic farms, and farms with less than 50 cows. Knowing that we will not be able to enroll a large number of farms, we decided to exclude types of farms that would represent “minorities” and could potentially skew our results; a focus on types of farms more representative of the majority of UK dairy farms was deemed a better approach for our study. All methods used to recruit farms and to collect data were approved by the University of Liverpool Veterinary Research Ethics Committee (Reference Number: VREC349). Each farm was visited once and visits were carried out across England and Wales from September 2015 to December 2016; the same trained researcher performed all measures.

### Lameness Assessment

All cows within the milking herd were mobility scored as they exited the parlor after a morning, afternoon, or evening milking by the same trained researcher (BG). The AHDB mobility scoring system was used (Table 1) (31). One herd was mobility scored on entry to the milking parlor. The proportion of clinically and severely lame cows was calculated for each farm. This within herd prevalence of lameness at the day of our visit was the main outcome of this study.

**Table 1:** Mobility scoring system as described by the Agriculture and Horticulture Development Board (31) and used in our study.

Score	Description
0	Walks with even weight bearing and rhythm on all four feet, with a flat back. Long, fluid strides possible

1	Steps uneven (rhythm or weight bearing) or strides shortened; affected limb or limbs not immediately identifiable

2	Uneven weight bearing on a limb that is immediately identifiable and or/obviously shortened strides (usually with an arch to the center of the back)

3	Unable to walk as fast as a brisk human pace (cannot keep up with the healthy herd) and signs of score 2

### Farm Risk Assessment

Information regarding farm characteristics, facility design, and management practices were collected by direct observation and through an interview with the farm manager. A detailed description of how information regarding each recorded variable was collected is presented in Table S1 in Supplementary Material.

#### Farm Characteristics

Milking herd size was calculated from the dataset used to record the mobility scores and also obtained from the farmer. Information regarding milk yield, calving pattern, breed, milking frequency, and age at first calving was obtained by interview with the farm manager. The number of milking groups was recorded after direct observation.

#### Facility Design

Collecting yard stocking density, total standing time per day during milking, and the number of cows per cluster were calculated. Standing times within a whole day would potentially be more useful to calculate, but this was deemed impractical, since the researcher could only spend a limited amount of time at each farm. Information regarding milking parlor type, the presence of mats within the milking parlor, collecting yard flooring type, and collecting yard groove pattern were obtained through interview with the farm manager and direct observation. Collecting yard grooving measurements and information regarding the presence of slopes, steps, or sharp corners in the collecting yard and the area around the milking parlor exit were collected through direct observation. The cleanliness of the collecting yard was subjectively assessed by the researcher and a 0–5 score was assigned, with 0 being extremely clean and 5 being extremely unclean. The collecting yard was checked for the presence of skid marks and the milking parlor exit width was measured.

#### Housing

All measurements undertaken were replicated for the milking cow group, dry cow group, transition cow group, and youngstock. The housing type (cubicles/loose/mixed), groove description, and measurements were collected by direct observation. Information on bedding depth and type together with an assessment of the abrasive nature of the bedding was collected through direct observation. A description of the resting area type (combining information on type of cubicles or use of loose housing with bedding depth) was also recorded. Cubicle stocking density was calculated for each pen, with the cubicle dimensions and cubicle cleanliness also evaluated. The total water trough length per cow and the feed fence length per cow were calculated. The feed fence type, feed fence height, and the neck rail height were collected by direct observation. The mattress or mat age was estimated by the farm manager, during interview.

#### Cow Track Features

Track quality, the presence of sharp stones, track material, track width (cm), and track width per cow (cm) were all collected by direct observation.

#### Diet

Feed type, feed energy levels, whether concentrates were fed within the parlor (or in out of parlor feeders), amount of concentrates fed per kg milk, amount of access to grazing (acres per cow), and protein content of the diet were noted after interview with the farm manager.

#### Management Practices

Information regarding the frequency of yards and passageway scraping, the method of scraping (manually, automated, or both), the frequency cubicles were cleaned, and fresh bedding applied was collected through interview with the farm manager. Whether the youngstock were routinely footbathed and routinely foot trimmed, and the cubicle quality of the youngstock were recorded following interview with the farm manager. The length of the transition period, whether fresh cows were separated and for how long, whether a straw yard was available to sick and lame cows, and if there was any shared grazing with other species were all recorded following interview with the farmer.

#### Lameness Management

Information regarding the frequency of claw trimming, whether early lactation (60–100 days post-calving) claw trims were undertaken, whether the farm recorded lesions, who was undertaking the claw trimming, the method, and whether the foot trimmer was trained were all collected following interview with the farm manager. Whether farms routinely mobility scored, the frequency, and who undertakes the mobility scoring were all recorded. Information regarding use of footbaths, the frequency of footbathing both in the summer and in the winter, whether the footbath featured a step down, and if cows were required to use the footbath when not in use, was recorded. Whether the footbath was permanent or temporary, the substrate used and the footbath dimensions were also recorded.

### Statistics

Statistical analysis was undertaken using JMP Pro 11.0.0 Software. The experimental unit was the farm. The outcome variable was the proportion of clinically lame cows (score ≥2) within the milking herd (within herd lameness prevalence). This was logit transformed to meet the assumptions of normality and to ensure estimates, and confidence intervals were correctly calculated. A number of measurements taken were not analyzed due to either a lack of variability or because of large proportions of missing data. The variables that were not analyzed were the ones associated with cubicles dimensions in the dry and transition cows’ sheds (most of the visited farms were using loose housing for dry and transition cows) and the type of the collecting yard flooring (lack of variability). Most continuous variables, such as herd size, milk yield, and age at first calving were also split into quartiles, resulting in categorical variables with four levels. A different grouping approach was employed for the variable related to collecting yard grooving spacing which was grouped into two categories (below or above 2 cm) as this was found to be a more biologically meaningful grouping approach.

Initially, univariable analysis (Parametric ANOVA test for categorical variables, linear regression for continuous variables) was performed to evaluate crude associations between explanatory variables and the herd lameness prevalence (logit transformed). Univariable analysis was also performed for grouped continuous variables. Univariable analysis was, therefore, undertaken for both grouped and ungrouped variables, and the variable with the lowest *P* value was chosen for use in the multivariable analysis. If analyses of grouped continuous variables were to reveal a non-linear relationship with lameness prevalence this would also have been taken into consideration. This, for example, would have been the case for a factor, where both extremes were unfavorably associated with lameness prevalence while intermediate values were more favorably associated with lameness prevalence. Associations between explanatory variables were also investigated to identify collinearity between variables. Variables with a *P* ≤ 0.20 in the univariable analysis were offered to a multivariable linear regression model. The model was built using a forward stepwise selection process. Only variables with a *P* < 0.1 were kept in the final model. The “informative missing” option in JMP Pro was used; this allows variables with missing values to be kept in the multivariable analysis. When two variables displayed collinearity, the variable with the highest *R*^2^ in a model with the outcome “lameness prevalence” was chosen. The Akaike’s information criterion was used to evaluate the models’ goodness of fit and the residuals were plotted to verify normality and homogeneity of variance. Model generated, adjusted means (least square means) and confidence intervals were back-transformed and presented as the percentage of clinically lame cows within the milking herd.

## Results

The median number of milking cows per herd was 193, ranging from 74 to 1,519 cows. Farms were enrolled from the South West (*n* = 34) and North West (*n* = 12) of England, Wales (*n* = 8), West Midlands (*n* = 6), and Yorkshire (*n* = 1). The median annual milk yield was 8,800 l with a range from 4,000 to 12,200 l. Forty-four of the study farms calved all year round, with 17 farms block-calving.

The mean within farm lameness prevalence was 31.6% with a SD of 13.9% and a range of 5.8–65.4%. In total, 14,700 cows were mobility scored with 4,145 cows found to be lame (28.19%); 536 cows were scored 3 (representing 3.65% of scored cows). Repeatability of mobility scoring by the investigator was assessed by scoring one herd (189 milking cows) twice on the same day. Within herd lameness prevalence was found to be 27.5 and 28% (morning and afternoon milking respectively) and this was deemed an acceptable level of repeatability.

Descriptive statistics of categorical variables detailing 33 farm characteristics and management practices, with the number of study farms in each category are presented in Table S2 in Supplementary Material; descriptive statistics of 11 continuous variables are presented in Table S3 in Supplementary Material.

### Univariable Analysis

At the univariable level, within farm lameness prevalence was statistically significantly (*P* < 0.05) associated with a number of variables. These results are presented in Tables 2 and 3. Variables not statistically significantly (*P* ≥ 0.05) associated with lameness prevalence, but with a *P* value below 0.2 was also offered in the multivariable analysis. These variables were: the frequency of footbathing in the summer, calving pattern, water trough length per animal (youngstock and milking herd), breed, track width, milking herd housing type, track quality, person undertaking mobility scoring, presence of sharp corners on exit of the milking parlor, frequency of preventative claw trimming, footbath width, milking herd feed fence neck rail height, transition herd housing type, milking parlor exit width, amount of concentrates fed in the parlor per kg of produced milk, feed fence type (youngstock), frequency of application of fresh bedding on cubicles, claw trimming method, and housing of sick cows in a different area. Both season and year of the visit were tested at the univariable level, and were not found to be statistically significantly associated with lameness prevalence.

**Table 2:** Categorical variables statistically significantly associated with lameness prevalence at the univariable analysis level in a study on 61 UK dairy herds in England and Wales.

Explanatory variable	Levels	*N*	Back Transformed mean	*P* value
Dry herd feed fence type	Feed fence on the floor	21	0.23	>0.001
	Self-feed fence	2	0.23	
	Trough	15	0.41	

Milking herd resting area type	Deep bedded (includes straw yards)	7	0.14	0.003
	Mats with shallow bedding	27	0.33	
	Mattresses with shallow bedding	17	0.34	
	Mixed	5	0.31	
	Concrete cubicles with shallow bedding	5	0.28	

Milking herd cleanliness of cubicles (0 is very clean and 5 is very unclean)	0	20	0.22	0.003
	1	13	0.35	
	2	10	0.35	
	3	2	0.45	

Milking herd bedding depth and type	Deep sand	3	0.09	0.003
	Deep straw	3	0.15	
	Deep wood pulp	1	0.33	
	Mixed	3	0.34	
	Shallow paper pulp	1	0.26	
	Shallow sand	3	0.40	
	Shallow sawdust	31	0.31	
	Shallow straw	16	0.34	

Collecting yard grooving spacing width	Up to 2 cm	35	0.37	>0.001
	2 cm and aboveNo grooving	113	0.23 0.22	

Youngstock cubicle quality	Poor	8	0.41	0.007
	Good	17	0.25	
	Excellent	2	0.10	

Frequency of footbathing undertaken in winter	Below once a week	10	0.22	0.012
	Between once and three times a week	22	0.38	
	Between four and six times a week	14	0.28	
	Above six times a week	13	0.26	

Is early lactation (60–100 DIM) preventative foot trimming undertaken?	Yes	14	0.22	0.012
	No	38	0.33	

Milking frequency	Twice a day	53	0.32	0.014
	Three times a day	8	0.19	

Routine footbathing of weaned youngstock	Yes	6	0.19	0.036
	No	53	0.31	

Frequency of mobility scoring	Less than quarterlyGreater than quarterly	28 11	0.36 0.21	0.008
	No mobility scoring undertaken	21	0.26	

**Table 3:** Continuous variables statistically significantly associated with lameness prevalence at the univariable analysis level in a study on 61 UK dairy herds in England and Wales.

Explanatory variable	*N*	Estimate	*P* value
Total number of clusters in the milking parlor	61	−0.022	0.005
Milking herd size	61	−0.001	0.006
Distance between top and bottom divider loop in cubicles (cm)	39	−0.031	0.007
Total number of stalls in the milking parlor	61	−0.013	0.011
Milking herd feed fence barrier height	59	−0.023	0.018
Distance from top brisket locator to the angle of the lower divider rail (cm)	30	−0.014	0.015

### Multivariable Analysis

Variables retained in the final multivariable model were: the amount of concentrates fed within the parlor (Estimate: 0.99, *P* = 0.06), collecting yard groove spacing width, whether farms undertake an early lactation claw trimming, the frequency of footbathing in winter, and resting area type and explained 72% of the variation (*R*^2^ = 0.72). Adjusted means with confidence intervals and *P* values for the categorical variables retained in the final model are presented in Table 4. Plausible interaction terms were also offered to the model; none was found to be statistically significant or to improve model fit.

**Table 4:** Categorical and continuous risk factors associated with herd lameness prevalence with adjusted back-transformed means and 95% confidence intervals for each different level (for categorical variables) or parameter estimates (for continuous variables) in a study on 61 UK dairy herds in England and Wales.

Variable	Category	Adjusted mean	95% CI	*P* value
Milking herd resting area type	Deep bedded (includes straw yards)	12.1%	8.6–17%	0.013
	Mats with shallow bedding	21.8%	17.2–27.2%	
	Mattresses with shallow bedding	22.8%	17.7–28.9%	
	Mixed	21.8%	14.7–31.1%	
	Concrete with shallow bedding	16.9%	11–25.2%	

Collecting yard grooving spacing width	No grooving	17.5%	11–27.1%	<0.001
	Above 2 cm	14%	10–19.1%	
	Below 2 cm	28.3%	23.2–33.9%	

Footbathing frequency in the winter	Below once a week	16%	11.9–21.1%	<0.001
	One to three times a week	26.4%	21–32.6%	
	Four to six times a week	20.8%	16.1–26.5%	
	Above six times a week	15.4%	11.2–20.7%	

Is early lactation (60–100 DIM) preventative foot trimming undertaken?	No	23.3%	19.5–27.7%	0.002
	Yes	14%	10.2–18.8%	

Concentrates in the parlor; how much per kg of milk (kg)?	Continuous variable	Estimate		0.06
		0.99		

*Results presented here were obtained from multivariable regression analysis*.

## Discussion

This cross-sectional study provides an update on lameness prevalence and identifies farm level risk factors in 61 farms across England and Wales. The lameness prevalence data presented within this study, suggest a decrease in UK within farm lameness prevalence, from 36.8 to 31.8% (6). Several risk factors were associated with the prevalence of lameness on study farms. A limitation of this cross-sectional study is that causal inferences cannot be made; in fact, lameness prevalence may itself impact on some of the explanatory variables. The visited farms were not randomly selected and eventually a relatively small number of farms were only visited once. 63 of the initially approached 124 farms decided not to participate in our study. The reasons for this decision were not investigated and, therefore, we cannot predict how this has potentially affected our prevalence estimates. For these reasons, external reproducibility and representativeness of our study may be reduced. On the other hand, a major strength of this study is that all farm measurements and mobility scorings were undertaken by one trained researcher.

The mean within farm lameness prevalence recorded in this study was 31.8%. The range in farm level prevalence found in this study (5.79–65.36%) was smaller than the range found in the Barker et al. study (0–79.2%) (6). A similar mobility scoring system was used in both studies, with the entire milking herd scored. Cows in the Barker et al. (6) study were mobility scored by multiple observers and scored on exit of the milking parlor or in the loafing yard, compared to one observer in our study. Interestingly, a similar, independent study conducted in the UK (using different farms) at the same time period with our study and using the same mobility scoring technique reported results very similar to ours; within herd lameness prevalence was 30%, ranging from 7 to 61% (32). Worldwide, these results are higher than the results presented by Cook (10) (within farm lameness prevalence of 21.1% in the summer and 23.9% in the winter) for farms sampled in Wisconsin (10). Multiple observers undertook the mobility scoring at different time points, and a different, but similar, mobility scoring system was used. Chapinal et al. (33) reported a high within farm lameness prevalence in North Eastern United States (54.8%); prevalence was lower in British Columbia (27.9%) and California (30.8%). Unfortunately, there is no widely available objective measure of cattle lameness. The current method uses mobility scoring as a means of identifying lame cows. Various scoring systems are undertaken in research (34–36); therefore, care must be taken when comparing lameness prevalence from different studies due to observer subjectivity and the scoring system used.

We show here that deep bedded cubicles or straw yards were associated with lower prevalence of lameness. This finding is supported by work undertaken by Ito et al., Husfeldt and Endres, and Chapinal et al. (33, 37, 38). Deep bedding is thought to be a more comfortable lying surface; this in turn increases the lying times of the cows (37, 39), reduces the time spent standing on hard wet surfaces (40), and ultimately reduces the risk of lameness. This was highlighted by Dippel et al. (41)who reported a decreased risk of lameness with increased lying comfort associated with deep bedded cubicles. The risk of digital dermatitis was also elevated in those farms using less than 5 cm in bedding compared to those farms using more (13). We also show here that deep sand was the bedding type associated with the lowest herd lameness prevalence (significant at the univariable analysis level).

Early lactation preventative claw trimming was associated with decreased lameness prevalence. This is an increasingly popular management practice within UK dairy farms, but as yet there have been no studies examining this practice during the early lactation period. There have been several studies showing routine claw trimming to be beneficial (23, 42), with studies showing a beneficial effect when increasing the frequency of claw trimming to twice annually (25, 26, 43). There have also been studies showing farms that perform trimming only when the claw is overgrown or when lameness has occurred have higher levels of lameness (25); a similar association at the univariable level was found here, however, due to the high correlation between routine claw trimming frequency and whether 60- to 100-day post calving checks were undertaken, claw trimming frequency was not kept in the final model. The basis behind this early lactation preventative claw trim is due to evidence showing that cows are most at risk from a lameness event for 5–7 months after calving, with an increased risk from white line disease 3–5 months into lactation (11, 44), with sole horn overgrowth a precursor to sole bruising and sole ulcers (45). It has been shown by Solano et al. (8) that overgrown claws result in cows being 1.4–1.7 times more likely to be lame, while a difference in claw height between the medial and lateral claw results in cows with an increased risk of lameness (42).

Several studies have shown that routine footbathing is associated with lower lameness prevalence (23, 46), and that increasing the frequency of footbathing decreases the risk further (33). We show here that farms that used a footbath at increased frequencies had reduced lameness prevalence. Interestingly, those farms that used a footbath less than once a week (including farms which did not use a footbath at all) also exhibited lower lameness prevalence than those farms that used a footbath only moderately. It could be hypothesized that because footbathing is the main control method for digital dermatitis (47), farmers using a footbath do so because they have digital dermatitis within their herds, and this could explain why farms that do not footbath have lower lameness prevalence than those only using a footbath 1–3 times a week. When footbathing was undertaken, increasing the frequency of footbathing was associated with reduced lameness. Farms that undertake footbathing, but not very frequently may have a digital dermatitis problem, however, the problem is not well controlled. Farms that very frequently footbath could be considered more proactive at controlling digital dermatitis.

We show here that larger spacing between grooves in the collecting yard was associated with lower lameness prevalence. Barker et al. (11) found that solid grooved concrete increased the risk of digital dermatitis; it was suggested that the grooving allowed an accumulation of slurry to remain on the flooring, acting as a reservoir for bacteria, even after scraping had been undertaken. Flooring that did not allow an accumulation of slurry was associated with decreased levels of digital dermatitis (13). When the gap between grooving is wider, scraping of the yards may be more effective, reducing the accumulation of slurry and, therefore, reducing the risk of lameness. Barker et al. (11) found that grooved flooring is associated with a twofold increased risk of white line disease, the paper explains that worn grooving is slippery, which is a risk factor for lameness, as described by Solano et al. (8) and Faull et al. (48). A further study by Pérez-Cabal and Alenda (23) found that grooved concrete increased the risk of lameness compared to solid concrete flooring. Therefore, a wider gap in the grooving may be associated with flooring that is less slippery and, therefore, more effective at providing grip than smaller shallower grooving.

Feeding larger amounts of concentrates in the parlor or in out of parlor feeders was associated with a tendency for greater prevalence of lameness. It has been shown that a fast rise in concentrates to the maximum amount was strongly, positively associated with digital dermatitis compared to a smaller step up in amount (13). Furthermore, dairy cattle with a high genetic merit for milk yield have been shown to have an increased risk of lameness (49, 50), with high producing cows requiring increased amounts of concentrates during the early lactation period. Additionally, feeding of larger amounts of concentrates could be predisposing cows to subacute ruminal acidosis which in turn has been associated with increased lameness risk (51).

## Conclusion

The results of this study have provided an update on the UK within herd lameness prevalence. It seems that lameness prevalence has been reduced within UK herds in the past 10 years; however, it should still be a significant concern to the dairy industry given the severe and wide range of effects as well as the still high prevalence. The wide range of prevalence between farms indicates that it is possible for farmers to achieve low levels of lameness prevalence in their herds. This study has confirmed the importance of some previously described risk factors for lameness, such as bedding depth or footbathing frequency. The association between early lactation foot trimming and reduced lameness prevalence is, to the best of our knowledge, reported for the first time.

## Ethics Statement

All methods used to recruit farms and to collect data were approved by the University of Liverpool Veterinary Research Ethics Committee (Reference Number: VREC349).

## Author Contributions

BG—conducted the field study, analyzed the data, and wrote the manuscript draft. DW—assisted statistical analysis of the data, and critically evaluated the manuscript. GO—corresponding author. Conceived and designed the study, assisted statistical analysis of the data, and critically evaluated the manuscript. All authors approved the final version of the paper and agree to be accountable for all aspects of the work.

## Conflict of Interest Statement

The authors declare that the research was conducted in the absence of any commercial or financial relationships that could be construed as a potential conflict of interest.
