# Supplementary material for: A Cross-Sectional Study Into the Prevalence of Dairy Cattle Lameness and Associated Herd-Level Risk Factors in England and Wales
Source: Front Vet Sci. 2018 Apr 5;5:65. doi: 10.3389/fvets.2018.00065 (PMC5895762; doi:10.3389/fvets.2018.00065)
Supplement: Supplementary file 1 [file table_1.docx]

Table S1. Description of the information collected during farm visits in a study on 61 UK dairy herds in England and Wales.

| Variable |  | Level | Explanation |
| --- | --- | --- | --- |
| Season | Categorical | Autumn | According to when the visit was made. |
|  |  | Winter |  |
|  |  | Summer |  |
| Year | Categorical | 2015 | According to when the visit was made. |
|  |  | 2016 |  |
| Year/season | Categorical | Autumn 2015 | According to when the visit was made. |
|  |  | Winter 2015 |  |
|  |  | Summer 2016 |  |
|  |  | Autumn 2016 |  |
| Herd size (number of cows) | Continuous |  | Obtained through interview with the farm manager. |
| Milking herd size (number of cows) | Continuous |  | Obtained through interview with the farm manager. |
| Lactation milk yield (kg) | Continuous |  | Obtained through interview with the farm manager. |
| Calving pattern | Categorical | All year round | Obtained through interview with the farm manager. |
|  |  | Block |  |
| Breed | Categorical | Holstein  Holstein Friesian  Other | Obtained through interview with the farm manager. |
| Number of milking groups | Categorical |  | Obtained through interview with the farm manager, and through direct observation. |
| Maximum group size for milking groups | Continuous |  | Obtained through direct observation. |
| Age at first calving (days) | Continuous |  | Obtained through interview with the farm manager. |
| Milking frequency | Categorical |  | Obtained through interview with the farm manager. |
| Collecting yard area (m^2^) | Continuous |  | Directly measured using a laser measuring device. |
| Collecting yard stocking density | Continuous |  | Calculated by dividing the collecting yard area by the maximum group size at milking. |
| Total standing time per day (per milking group, in minutes) | Continuous |  | Calculated as the duration from when the first cow in a group enters the milking parlour to when the last cow in this group exits multiplied by the milking frequency. Group with the highest value from each farm was included in statistical analyses. |
| Number of stalls in the milking parlour | Continuous |  | Obtained by direct observation. |
| Number of clusters in the milking parlour | Continuous |  | Obtained by direct observation. |
| Number of cows per cluster | Continuous |  | Calculated by dividing group size by the number of clusters in the milking parlour. |
| Parlour type | Categorical | Rotary | Obtained by direct observation. |
|  |  | Herringbone |  |
|  |  | Rapid exit |  |
|  |  | Other |  |
| Presence of mats in the parlour | Binary | Yes | Obtained by direct observation. |
|  |  | No |  |
| Collecting yard flooring type | Categorical | Solid flooring | Obtained by direct observation. |
|  |  | Slatted flooring |  |
| Presence of grooved concrete in collecting yard | Binary | Yes | Obtained by direct observation. |
|  |  | No |  |
| Collecting yard groove pattern | Categorical | Square | Obtained by direct observation. |
|  |  | Linear |  |
|  |  | Hexagonal |  |
|  |  | Mixed |  |
| Collecting yard grooving width (cm) | Continuous |  | Directly measured using a laser measuring device. The measurement was taken from one side of the raised portion to the other. |
| Collecting yard grooving spacing width (cm) | Continuous |  | Directly measured using a laser measuring device. The measurement was taken of the grooving gap between raised portions in the concrete. |
| Presence of slopes or steps in the collecting yard and milking parlour | Binary | Yes | Obtained by direct observation. |
|  |  | No |  |
| Presence of sharp corners in the collecting yard | Binary | Yes | Obtained by direct observation. |
|  |  | No |  |
| Presence of sharp corners on exit of the milking parlour | Binary | Yes | Obtained by direct observation. |
|  |  | No |  |
| Collecting yard cleanliness | Categorical | 0 | Subjective assessment undertaken by the researcher, where 0 is very clean and 5 is very unclean. |
|  |  | 1 |  |
|  |  | 2 |  |
|  |  | 3 |  |
|  |  | 4 |  |
|  |  | 5 |  |
| Presence of skid marks in the collecting yard | Binary | Yes | Obtained by direct observation. |
|  |  | No |  |
| Is there a cooling system present within the milking parlour? | Binary | Yes | Obtained by direct observation. |
|  |  | No |  |
| Milking parlour exit width (cm) | Continuous |  | Directly measured using a laser measuring device. |
| Method of yard and passageway scraping | Categorical | Manual (tractor etc) | Obtained through interview with the farm manager. |
|  |  | Automatic scrapers |  |
|  |  | Both |  |
| Presence of automated scrapers | Binary | Yes | Obtained through interview with the farm manager, and through direct observation. |
|  |  | No |  |
| Frequency of yard and passageway scraping in summer | Continuous |  | Obtained through interview with the farm manager. |
| Frequency of yard and passageway scraping in winter | Continuous |  | Obtained through interview with the farm manager. |
| Milking herd housing type | Categorical | Cubicles | Obtained by direct observation. |
|  |  | Loose housing |  |
|  |  | Mixed housing |  |
| Milking herd grooving description | Categorical | Linear | Obtained by direct observation. |
|  |  | Square |  |
|  |  | Mixed |  |
| Milking herd: Resting area type | Categorical | Deep bedded (includes straw yards) | Obtained by direct observation and measurement. Bedding consisting of greater than 4 inches was considered deep bedding, shallow bedding consists of cubicles with only a small amount of substrate (for example straw or sawdust). Mixed means that different milking groups were housed on different cubicle types. |
|  |  | Mats with shallow bedding |  |
|  |  | Mattresses with shallow bedding |  |
|  |  | Concrete cubicles with shallow bedding |  |
|  |  | Mixed |  |
| Milking herd: Bedding type | Categorical | Deep sand | Obtained by direct observation. This consists of the type and amount of bedding substrate. Mixed means that there were different milking groups housed on different bedding substrates. |
|  |  | Deep straw |  |
|  |  | Deep wood pulp |  |
|  |  | Shallow sand |  |
|  |  | Shallow straw |  |
|  |  | Shallow sawdust |  |
|  |  | Shallow paper pulp |  |
|  |  | Mixed |  |
| Milking herd: Bedding abrasiveness | Categorical | Excellent | This test was used to subjectively assess the abrasiveness of the different bedding substrates on the skin of the researcher’s knuckles. |
|  |  | Good |  |
|  |  | Poor |  |
|  |  | Mixed |  |
| Milking herd: Total number of cubicles in milking herd housing | Continuous |  | Obtained by direct observation, only cubicles available for use (for example those that weren’t broken or those with no access) |
| Milking herd: Cubicles per cow | Continuous |  | This was calculated by dividing the total number of cubicles by the number of cows using that set of housing. |
| Milking herd:  Freestall width (cm) | Continuous |  | Directly measured using a laser measuring device. Measured from one freestall partition to the other at the height of the neck nail. |
| Milking herd:  Freestall height (cm) | Continuous |  | Directly measured using a laser measuring device. This was measured from the top of the freestall partition down to the surface of the freestall bedding. |
| Milking herd:  Total stall length (cm) | Continuous |  | Directly measured using a laser measuring device. This was measured from the rear curb to the wall of the housing, or if head to head cubicles were used then the middle bar supporting the freestall partitions. |
| Milking herd:  Divider loop internal diameter (cm) | Continuous |  | Directly measured using a laser measuring device. This was measured from the bottom of the higher divider rail in the freestall partition to the top of the lower divider rail. |
| Milking herd:  Type of brisket board | Categorical | None | Obtained by direct observation. Straight refers to boards which project upwards with corners such as wooden boards. Rounded refers to boards with rounded characteristics such as polythene pipes. |
|  |  | Straight |  |
|  |  | Rounded |  |
| Milking herd:  Length from wall to brisket board (cm) | Continuous |  | Directly measured using a laser measuring device. This is the length from the wall (or if head to head cubicles were used then the middle bar supporting the freestall partitions) to the mid-point of the brisket board. |
| Milking herd:  Length from rear curb to neck rail (cm) | Continuous |  | Directly measured using a laser measuring device. This is measured from the neck rail to the rear curb on the horizontal axis. |
| Milking herd:  Height of neck rail above bedding surface (cm) | Continuous |  | Directly measured using a laser measuring device. This is measured from the underside of the neck rail to the bedding surface. |
| Milking herd:  Distance from stall surface to the lower divider rail (cm). | Continuous |  | Directly measured using a laser measuring device. Measured from the stall surface to the bottom of the lower divider rail. |
| Milking herd:  Distance from brisket locator to angle of the lower divider rail (cm) | Continuous |  | Directly measured using a laser measuring device. Measured from the angle of the lower divider rail to the brisket locator. |
| Milking herd:  Diagonal distance from neck rail to rear curb (cm) | Continuous |  | Directly measured using a laser measuring device. Measured on the diagonal axis, from the neck rail to the rear curb. |
| Milking herd:  Kerb height (Without mattress/mats, cm) | Continuous |  | Directly measured using a laser measuring device. From the top of the concrete stall base to the passageway surface. |
| Milking herd:  Kerb height (including mattress/mats, cm) | Continuous |  | Directly measured using a laser measuring device. From the top of the mat or mattress to the passageway surface. |
| Milking herd:  Cleanliness of cubicles | Categorical | 0 | Subjective assessment undertaken by the researcher, where 0 is very clean and 5 is very unclean. |
|  |  | 1 |  |
|  |  | 2 |  |
|  |  | 3 |  |
|  |  | 4 |  |
|  |  | 5 |  |
| Milking herd:  Cubicle bedding frequency | Ordinal |  | Obtained through interview with the farm manager. |
| Milking herd:  Cubicle cleaning frequency | Ordinal |  | Obtained through interview with the farm manager. |
| Milking herd:  Total water trough length (cm) | Continuous |  | All water troughs of which the milking cows have access were directly measured using a laser measuring device. These lengths were added together to calculate the total water trough length. |
| Milking herd:  Water trough length per cow | Continuous |  | This was calculated by dividing the milking herd total water trough length by the maximum number of milking cows. |
| Milking herd:  Feed fence type | Categorical | Floor | Obtained by direct observation. Other feed fence types consisted of self-feeding systems or ring feeders. |
|  |  | Trough |  |
|  |  | Mixed |  |
|  |  | Other |  |
| Milking herd:  Feed fence total length (cm) | Continuous |  | All feed fences of which the milking cows have access were directly measured using a laser measuring device. These lengths were added together to calculate the total feed fence length. |
| Milking herd:  Feed fence length per cow (cm) | Continuous |  | This was calculated by dividing the milking herd total feed fence length by the maximum number of milking cows. |
| Milking herd:  Feed fence barrier height (cm) | Continuous |  | Directly measured using a laser measuring device. Measured from the passageway surface to the highest point of the barrier. |
| Milking herd:  Feed fence neck rail height (cm) | Continuous |  | Directly measured using a laser measuring device. From the underside of the neck rail to the top of the feed fence barrier. |
| Milking herd:  Mat or mattress age | Continuous |  | Obtained through interview with the farm manager. |
| Milking herd: Loose housing lying area (m^2)^ | Continuous |  | Directly measured using a laser measuring device. |
| Milking herd: Number of cows in loose housing | Continuous |  | Obtained by direct observation. |
| Milking herd: Loose housing density (m^2^/cow) | Continuous |  | Calculated by dividing the loose housing area by the milking group size. |
| Milking herd:  Track Quality | Categorical | 0 | Subjective assessment undertaken by the researcher, where 0 is the best quality, clean with good grip and without the presence of large or sharp stones. Whereas five is the worst quality, being excessively muddy, damaged concrete or slippery with large or sharp stones. |
|  |  | 1 |  |
|  |  | 2 |  |
|  |  | 3 |  |
|  |  | 4 |  |
|  |  | 5 |  |
| Presence of sharp stones/gravel on the track | Binary | Yes | Subjective assessment undertaken by the researcher. |
|  |  | No |  |
| Track material | Categorical | Astroturf | Obtained through interview with the farm manager. |
|  |  | Concrete |  |
|  |  | Earth |  |
|  |  | Hardcore |  |
|  |  | Road |  |
|  |  | Woodchip |  |
|  |  | Mixed |  |
| Track width (cm) | Continuous |  | Directly measured using a laser measuring device measuring the width of track material. |
| Track width per cow (cm/cow) | Continuous |  | Calculated by dividing the track width by the maximum milking group size. |
| Milking herd:  Feed type (summer) | Categorical | Total mixed ration | Obtained through interview with the farm manager. |
|  |  | Semi-total mixed ration |  |
|  |  | Grass based |  |
| Milking herd:  Feed type (winter) | Categorical | Total mixed ration | Obtained through interview with the farm manager. |
|  |  | Semi-total mixed ration |  |
|  |  | Grass based |  |
| Milking herd:  Diet energy levels | Categorical | High | Obtained through interview with the farm manager. |
|  |  | Medium |  |
|  |  | Low |  |
| Milking herd:  Are concentrates fed in the parlour (or in out of parlour feeders)? | Binary | Yes | Obtained through interview with the farm manager. |
|  |  | No |  |
| Milking herd:  Concentrates in the parlour; how much per kg of milk (kg)? | Continuous |  | Obtained through interview with the farm manager. |
| Milking herd:  Protein content of the diet (% of dry matter) | Continuous |  | Obtained through interview with the farm manager. |
| Are weaned youngstock footbathed routinely? | Binary | Yes | Obtained through interview with the farm manager. |
|  |  | No |  |
| Are weaned youngstock claw trimmed routinely? | Binary | Yes | Obtained through interview with the farm manager. |
|  |  | No |  |
| What is the quality of the weaned youngstock cubicles? | Binary | Yes | Obtained through interview with the farm manager. |
|  |  | No |  |
| Claw trimming:  Are lesions recorded? | Binary | Yes | Obtained through interview with the farm manager. |
|  |  | No |  |
| Claw trimming:  Who undertakes the claw trimming? | Categorical | In house | Obtained through interview with the farm manager. |
|  |  | Paid contractor |  |
|  |  | Both |  |
| Claw trimming:  Method used? | Categorical | Dutch | Obtained through interview with the farm manager. |
|  |  | Dairyland |  |
|  |  | No standardised method |  |
| Claw trimming:  Are the trimmers qualified? | Binary | Recent training undertaken | Obtained through interview with the farm manager. |
|  |  | No recent training undertaken |  |
| Claw trimming:  Is preventative claw trimming undertaken? | Binary | Yes | Obtained through interview with the farm manager. |
|  |  | No |  |
|  |  |  |  |
| Claw trimming:  Are claws trimmed during the early lactation period (60-100d post calving)? | Binary | Yes | Obtained through interview with the farm manager. |
|  |  | No |  |
| Claw trimming:  Frequency of preventative claw trimming a year | Categorical | No routine claw trimming undertaken | Obtained through interview with the farm manager. The number of times per lactation cycle a cow will receive routine preventative claw trimming. |
|  |  | Once a year claw trimming (At drying off) |  |
|  |  | Twice a year claw trimming (At drying off and early lactation) |  |
| Is mobility scoring undertaken routinely? | Binary | Yes | Obtained through interview with the farm manager. |
|  |  | No |  |
| Frequency of mobility scoring a year | Continuous |  | Obtained through interview with the farm manager. |
| Person undertaking mobility scoring | Categorical | Farm staff | Obtained through interview with the farm manager. |
|  |  | Independent mobility scorer |  |
|  |  | Veterinary surgeon |  |
| Is footbathing undertaken? | Binary | Yes | Obtained through interview with the farm manager. |
|  |  | No |  |
| Is there a step down into the footbath? | Binary | Yes | Obtained through interview with the farm manager. |
|  |  | No |  |
| Do cows walk through the footbath when not in use? | Binary | Yes | Obtained through interview with the farm manager and observed by the researcher. |
|  |  | No |  |
| Footbath type | Categorical | Permanent | Obtained by direct observation. |
|  |  | Temporary |  |
| Footbathing frequency (summer, times/week) | Continuous |  | Obtained through interview with the farm manager. |
| Footbathing frequency (winter, times/week) | Continuous |  | Obtained through interview with the farm manager. |
| Footbath substrate | Categorical | Copper Sulphate based | Obtained through interview with the farm manager. Other includes products such as detergents and organic acids. |
|  |  | Formalin and Copper Sulphate |  |
|  |  | Formalin based |  |
|  |  | Other |  |
| Footbath length (cm) | Continuous |  | Directly measured using a laser measuring device. |
| Footbath width (cm) | Continuous |  | Directly measured using a laser measuring device. |
| Footbath depth (cm) | Continuous |  | Directly measured using a laser measuring device. |
| Transition cow length (weeks) | Continuous |  | Obtained through interview with the farm manager. |
| Are fresh cows separated? | Binary | Yes | Obtained through interview with the farm manager. |
|  |  | No |  |
| Length of fresh cow separation (days) | Continuous |  | Obtained through interview with the farm manager. |
| Are sick cows housed in a hospital group? | Binary | Yes | Obtained through interview with the farm manager. |
|  |  | No |  |
| Is a straw yard available for sick cows, even if there is no hospital group? | Binary | Yes | Obtained through interview with the farm manager. |
|  |  | No |  |
| Incidence of SARA or rumenal acidosis? | Binary | Yes | Obtained through interview with the farm manager. |
|  |  | No |  |
| Have you previously been involved in the DairyCo healthy feet programme? | Binary | Yes | Obtained through interview with the farm manager. |
|  |  | No |  |
| Any shared grazing with other species? | Binary | Yes | Obtained through interview with the farm manager. |
|  |  | No |  |
| Transition and dry herd housing type | Categorical | Cubicles housing | Obtained by direct observation. |
|  |  | Loose housing |  |
|  |  | Turned out/loose housing |  |
|  |  | Turned out |  |
| Transition and dry herd grooving description | Categorical | Linear | Obtained by direct observation. |
|  |  | Square |  |
|  |  | Mixed |  |
| Transition and dry herd: Bedding depth | Categorical | Deep | Obtained by direct observation and measurement. Beddings consisting of greater than 4 inches was considered dep bedding, shallow bedding consists of cubicles with only a small amount of substrate (for example straw or sawdust). |
| Transition and dry herd: Bedding abrasiveness | Categorical | Excellent | This was used to subjectively assess the abrasiveness of the different bedding substrates on the skin of the researcher’s knuckles. |
|  |  | Good |  |
|  |  | Poor |  |
|  |  | Mixed |  |
| Transition and dry herd: Cubicles per cow | Continuous |  | This was calculated by dividing the total number of cubicles by the number of cows using that set of housing. |
| Transition and dry herd:  Cubicles width (cm) | Continuous |  | Directly measured using a laser measuring device. Measured from one freestall partition to the other at the height of the neck nail. |
| Transition and dry herd:  Cubicles height (cm) | Continuous |  | Directly measured using a laser measuring device. This was measured from the top of the freestall partition down to the surface of the freestall. |
| Transition and dry herd:  Total stall length (cm) | Continuous |  | Directly measured using a laser measuring device. This was measured from the rear curb to the wall of the housing, or if head to head cubicles were used then the middle bar holding the freestall partitions. |
| Transition and dry herd:  Divider loop internal diameter (cm) | Continuous |  | Directly measured using a laser measuring device. This was measured from between the bottom of the higher divider rail in the freestall partition to the top of the lower divider rail. |
| Transition and dry herd:  Type of brisket board | Categorical | None | Obtained by direct observation. Straight refers to boards which project upwards with corners such as wooden boards. Rounded refers to boards with rounded characteristics such as polythene pipes. |
|  |  | Straight |  |
|  |  | Rounded |  |
| Transition and dry herd:  Length from wall to brisket board (cm) | Continuous |  | Directly measured using a laser measuring device. This is the length from the wall (or if head to head cubicles were used then the middle bar supporting the freestall partitions) to the mid-point of the brisket board. |
| Transition and dry herd:  Length from rear curb to neck rail (cm) | Continuous |  | Directly measured using a laser measuring device. This is measured from the neck rail to the rear curb on the horizontal axis. |
| Transition and dry herd:  Height of neck rail above bedding surface (cm) | Continuous |  | Directly measured using a laser measuring device. This is measured from the underside of the neck rail to the bedding surface. |
| Transition and dry herd:  Distance from stall surface to top of the lower divider rail (cm) | Continuous |  | Directly measured using a laser measuring device. Measured from the stall surface to the bottom of the lower divider rail. |
| Transition and dry herd:  Distance from brisket locator to angle of the lower divider rail (cm) | Continuous |  | Directly measured using a laser measuring device. Measured from the angle of the lower divider rail to the brisket locator. |
| Transition and dry herd:  Diagonal distance from neck rail to rear curb (cm) | Continuous |  | Directly measured using a laser measuring device. Measured on the diagonal axis, from the neck rail to the rear curb. |
| Transition and dry herd:  Kerb height (Without mattress/mats, cm) | Continuous |  | Directly measured using a laser measuring device. From the top of the concrete stall base to the passageway surface. |
| Transition and dry herd:  Kerb height (including mattress/mats, cm) | Continuous |  | Directly measured using a laser measuring device. From the top of the mat or mattress to the passageway surface. |
| Transition and dry herd:  Cleanliness of cubicles | Categorical | 0 | Subjective assessment undertaken by the researcher, where 0 is very clean and 5 is very unclean. |
|  |  | 1 |  |
|  |  | 2 |  |
|  |  | 3 |  |
|  |  | 4 |  |
|  |  | 5 |  |
| Transition and dry herd:  Frequency of cubicles bedding application (times/week) | Continuous |  | Obtained through interview with the farm manager. |
| Transition and dry herd:  Frequency of cubicles cleaning (times/week) | Continuous |  | Obtained through interview with the farm manager. |
| Transition and dry herd:  Total water trough length (cm) | Continuous |  | All water troughs of which the transition cows have access were directly measured using a laser measuring device. These lengths were added together to calculate the total water trough length. |
| Transition and dry herd:  Water trough length per cow (cm) | Continuous |  | This was calculated by dividing the transition herd total water trough length by the maximum number of transition cows. |
| Transition and dry herd:  Feed fence type | Categorical | Floor | Obtained by direct observation. |
|  |  | Trough |  |
|  |  | Round feeders |  |
|  |  | Self-feeding systems |  |
| Transition and dry herd:  Feed fence total length (cm) | Continuous |  | All feed fences of which the transition cows have access were directly measured using a laser measuring device. These lengths were added together to calculate the total feed fence length. |
| Transition and dry herd:  Feed fence length per cow (cm) | Continuous |  | This was calculated by dividing the transition herd total feed fence length by the maximum number of transition cows. |
| Transition and dry herd:  Feed fence barrier height (cm) | Continuous |  | Directly measured using a laser measuring device. Measured from the passageway surface to the top of the highest point of the barrier. |
| Transition and dry herd:  Feed fence neck rail height (cm) | Continuous |  | Directly measured using a laser measuring device. From the underside of the neck rail to the top of the feed fence barrier. |
| Transition and dry herd:  Mat or mattress age | Continuous |  | Obtained through interview with the farm manager. |
| Transition and dry herd: Loose housing lying area (m^2^) | Continuous |  | Directly measured using a laser measuring device. |
| Transition and dry herd: Number of cows in housing | Continuous |  | Obtained by direct observation. |
| Transition and dry herd: Loose housing density (m^2^/cow) | Continuous |  | Calculated by dividing the loose housing area by the maximum transition group size. |
| Youngstock: Housing type | Categorical | Freestall housing | Obtained by direct observation. |
|  |  | Loose housing |  |
|  |  | Turned out |  |
| Youngstock: Bedding comfort | Categorical | Excellent | Subjective assessment undertaken by the researcher. The bedding was tested for abrasiveness. Furthermore, the researcher assessed comfort by dropping to their knees whilst in the pen. |
|  |  | Good |  |
|  |  | Poor |  |
| Presence of skid marks in the youngstock housing | Binary | Yes | Obtained by direct observation. |
|  |  | No |  |
| Youngstock: Water trough type | Categorical | Automatic water dispenser | Obtained by direct observation. |
|  |  | Bucket |  |
|  |  |  |  |
| Youngstock:  Total water trough length (cm) | Continuous |  | The total water troughs within a pen of which the youngstock cows have access were directly measured using a laser measuring device. |
| Youngstock:  Water trough length per cow | Continuous |  | This was calculated by dividing the youngstock total water trough length by the number of youngstock within that pen. |
| Youngstock: Feed fence type | Categorical | Bucket | Obtained by direct observation. |
|  |  | Floor |  |
|  |  | Trough |  |
|  |  | None |  |
| Youngstock: Total feed fence length (cm) | Continuous |  | The total feed fence length within a pen of which the youngstock cows have access were directly measured using a laser measuring device. |
| Youngstock: Feed fence length per cow | Continuous |  | This was calculated by dividing the youngstock total feed fence length by the number of youngstock cows in a pen. |
| Youngstock: Feed barrier height (cm) | Continuous |  | Directly measured using a laser measuring device. Measured from the passageway surface to the top of the highest point of the barrier. |
| Youngstock: Loose housing lying area (m^2^) | Continuous |  | Directly measured using a laser measuring device. |
| Youngstock: Number of cows in pen | Continuous |  | Obtained by direct observation. |
| Youngstock: Loose housing density (m^2^/animal) | Continuous |  | Calculated by dividing the loose housing area by the youngstock group size. |
