# Supplementary material for: A Cross-Sectional Study Into the Prevalence of Dairy Cattle Lameness and Associated Herd-Level Risk Factors in England and Wales
Source: Front Vet Sci. 2018 Apr 5;5:65. doi: 10.3389/fvets.2018.00065 (PMC5895762; doi:10.3389/fvets.2018.00065)
Supplement: Supplementary file 2 [file table_2.docx]

Table S2. Descriptive statistics regarding farm characteristics and management practices in a study on 61 UK dairy herds in England and Wales.

| Explanatory Variables | Level | Percentage | Number of Farms |
| --- | --- | --- | --- |
| Breed | Holstein | 23.0% | 14 |
|  | Holstein Friesian | 44.3% | 27 |
|  | Mixed | 32.8% | 20 |
| Number of milking groups | One group | 55.7% | 34 |
|  | Two groups | 13.1% | 8 |
|  | Three groups | 14.8% | 9 |
|  | Four groups | 9.8% | 6 |
|  | Five and above groups | 6.6% | 4 |
| Milking frequency | Twice a day | 86.9% | 53 |
|  | Three times a day | 13.1% | 8 |
| Parlour type | Herringbone | 88.5% | 54 |
|  | Other (Including Rotary) | 3.3% | 2 |
|  | Rapid exit | 8.2% | 5 |
| Presence of mats in the parlour | Yes | 18.0% | 11 |
|  | No | 82.0% | 50 |
| Presence of grooved concrete in collecting yard | Yes | 90.2% | 55 |
|  | No  Missing information | 4.9% | 3  3 |
|  |  | 4.9% |  |
| Collecting yard grooving spacing width | Up to 2 cm  2 cm and above  No grooving  Missing Information | 57.4%  18%  4.9%  19.7% | 35  11  3  12 |
| Method of yard and passageway scraping | Automatic | 13.1% | 8 |
|  | Manual (Tractor) | 77.0% | 47 |
|  | Both | 9.8% | 6 |
| Milking Herd Housing Type | Cubicles | 93.4% | 57 |
|  | Loose | 4.9% | 3 |
|  | Mixed | 1.6% | 1 |
| Resting area type | Deep bedded (includes straw yards) | 11.5% | 7 |
|  | Mats with shallow bedding | 44.3% | 27 |
|  | Mattresses with shallow bedding | 27.9% | 17 |
|  | Mixed | 8.2% | 5 |
|  | Concrete cubicles with shallow bedding | 8.2% | 5 |
| Milking herd bedding depth and type | Deep sand | 4.9% | 3 |
|  | Deep straw | 4.9% | 3 |
|  | Shallow sand | 4.9% | 3 |
|  | Shallow sawdust | 50.8% | 31 |
|  | Shallow straw | 26.2% | 16 |
|  | Mixed | 4.9% | 3 |
|  | Deep wood pulp | 1.6% | 1 |
|  | Shallow paper pulp | 1.6% | 1 |
| Cubicle Bedding Frequency | Less than once a week | 36.1% | 22 |
|  | Once a week | 27.9% | 17 |
|  | More than once a week  No cubicles | 29.5%  6.6% | 18  4 |
| Cubicle Cleaning Frequency | Once a day | 14.8% | 9 |
|  | Twice a day | 65.6% | 40 |
|  | Three times a day  No cubicles | 13.1%  6.6% | 8  4 |
| Milking herd feed fence type | Feed fence on the floor | 47.5% | 29 |
|  | Trough | 37.7% | 23 |
|  | Mixed | 8.2% | 5 |
|  | Other (Including self-feeding systems)  Missing information | 4.9%  1.6% | 3  1 |
|  | No tracks | 16.4% | 10 |
| Track Material | Concrete (solid and sleepers) | 32.8% | 20 |
|  | Hard-core or crushed stone | 26.2% | 16 |
|  | Earth | 16.4% | 10 |
|  | Mixed | 3.3% | 2 |
|  | Other | 4.9% | 3 |
| Concentrates fed in the parlour or out of parlour feeders | Yes | 72.1% | 44 |
|  | No  Missing information | 26.2%  1.6% | 16  1 |
| Routine footbathing of weaned youngstock | Yes | 9.8% | 6 |
|  | No  Missing information | 86.9%  3.3% | 53  2 |
| Routine Claw trimming of weaned youngstock | Yes | 4.9% | 3 |
|  | No  Missing information | 91.8%  3.3% | 56  2 |
| Record lesions at claw Trimming | Yes | 80.3% | 49 |
|  | No | 19.7% | 12 |
| Person undertaking Claw Trimming | Paid contractor | 32.8% | 20 |
|  | In house | 41.0% | 25 |
|  | Both | 26.2% | 16 |
| Claw Trimming Method | Dutch method | 70.5% | 43 |
|  | Dairyland | 4.9% | 3 |
|  | No standardised method  Missing information | 16.4%  8.2% | 10  5 |
| Claw Trimming Training | Recent training undertaken | 90.2% | 55 |
|  | No recent training undertaken | 9.8% | 6 |
| Preventative Claw Trimming Undertaken | Yes | 63.6% | 39 |
|  | No  Missing information | 23%  13.1% | 14  8 |
| Is early lactation (60-100 DIM) preventative foot trimming undertaken? | Yes | 23% | 14 |
|  | No  Missing information | 62.3%  14.8% | 38  9 |
| Frequency of claw trimming | No routine claw trimming Undertaken | 23% | 14 |
|  | Once a year claw trimming | 44.3% | 27 |
|  | Twice a year claw trimming  Missing information | 19.7%  13.1% | 12  8 |
| Routine mobility scoring | Yes | 65.6% | 40 |
|  | No | 34.4% | 21 |
| Frequency of mobility scoring | Less than quarterly | 45.9% | 28 |
|  | Greater than quarterly | 18% | 11 |
|  | No mobility scoring undertaken  Missing information | 34.4%  1.6% | 21  1 |
| Person undertaking the mobility scoring | Farm Staff | 27.9% | 17 |
|  | Independent mobility scorer | 11.5% | 7 |
|  | Veterinary Surgeon | 6.6% | 4 |
| Routine footbathing of the milking herd | Yes | 90.2% | 55 |
|  | No | 9.8% | 6 |
| Footbath type | Permanent | 57.4% | 35 |
|  | Temporary  No footbath | 32.8%  9.8% | 20  6 |
| Footbath frequency in winter | Below once a week | 16.4% | 10 |
|  | Between once and three times a week | 36.1% | 22 |
|  | Between four and six times a week | 23% | 14 |
|  | Above six times a week  Missing information | 21.3%  3.3% | 13  2 |
| Footbath Frequency in Summer | Below once a week | 21.3% | 13 |
|  | Between once and three times a week | 37.7% | 23 |
|  | Between four and six times a week | 16.4% | 10 |
|  | Above six times a week  Missing information | 21.3%  3.3% | 13  2 |
| Footbath Substrate | Formalin | 52.5% | 32 |
|  | Copper sulphate | 9.8% | 6 |
|  | Both copper sulphate and formalin | 23% | 14 |
|  | Other | 4.9% | 3 |
