# Supplementary material for: A Cross-Sectional Study Into the Prevalence of Dairy Cattle Lameness and Associated Herd-Level Risk Factors in England and Wales
Source: Front Vet Sci. 2018 Apr 5;5:65. doi: 10.3389/fvets.2018.00065 (PMC5895762; doi:10.3389/fvets.2018.00065)
Supplement: Supplementary file 3 [file table_3.docx]

Table S3. Descriptive statistics regarding 11 continuous farm characteristics and management practices in a study on 61 UK dairy herds in England and Wales (continuous variables).

| Explanatory Variables | N | Mean | Median | Range |
| --- | --- | --- | --- | --- |
| Milking herd size | 61 | 239.9 | 193 | 74-1519 |
| Milking herd number of cubicles per cow | 51 | 1.1 | 1.1 | 0.6-1.7 |
| Milking herd water trough length per cow (cm) | 61 | 6.4 | 6.5 | 1.4-14.0 |
| Milking herd feed fence length per cow (cm) | 60 | 74 | 57 | 12-884 |
| Track Width (cm) | 52 | 423 | 405 | 210-930 |
| Milking herd protein content of the diet (% of dry matter) | 37 | 17.2 | 17 | 15.8-20.5 |
| Footbath Length (cm) | 51 | 323 | 300 | 130-636 |
| Footbath Width (cm) | 51 | 118 | 90 | 50-318 |
| Transition Group Length (weeks) | 59 | 2.75 | 3 | 0-8.6 |
| Total number of clusters in the milking parlour | 61 | 19.9 | 20 | 4-80 |
| Milking herd feed fence barrier height | 59 | 118.3 | 60 | 33-83.8 |
